# On the future of therapeutic vaccination in chronic hepatitis B

**DOI:** 10.2144/fsoa-2019-0104

**Published:** 2019-10-30

**Authors:** Julio Cesar Aguilar Rubido

**Affiliations:** 1 Head, Hepatitis B Therapeutic Vaccine Project, Center for Genetic Engineering & Biotechnology Ave. 31/158 & 190, Playa, Havana 10600, Cuba

**Keywords:** chronic hepatitis B, clinical trials, vaccination

According to the WHO, approximately 254 million patients are chronic carriers of hepatitis B virus (HBV) and the fatalities are in the range of 0.5 to 1 million patients per year [1]. Only a small fraction of patients requiring treatment are on therapy. Untreated patients are at risk of suffering liver cirrhosis and hepatocellular carcinoma.

Chronic hepatitis B (CHB) is one of the diseases where therapeutic vaccination has been tested with enthusiasm and hope, due to the limitations and low effectiveness [2] of current treatments. Although hepatitis B commercial vaccines are efficient in preventing HBV infection, they have failed in the therapeutic setting, leading to the development of more potent vaccine candidates.

Therapeutic vaccination can be conceptualized as the immunization strategy aimed at activating or modifying the immune system in order to cure, control or limit the progression of a specific pathological condition, using an active and specific stimulation of the immune system. This therapeutic approach has been attempted in the fields of chronic infectious diseases, cancer, autoimmunity, allergy and the treatment of addiction to several drugs.

The working hypothesis of therapeutic vaccination in the field of CHB is based on the understanding of the immunological profile of patients with natural- or treatment-induced serological and virological responses, in contrast to the profile induced in those unable to control HBV replication and the related liver damage. Mimicking this immune profile, several vaccine candidates were developed and tested. Even after the exciting Phase I/II results, several companies have stopped studies with their candidates since they failed to fulfill specific clinical end points in subsequent trials. A closer look into these clinical results is required.

Actions have been taken to improve therapeutic vaccines: inclusion of new antigens; the use of different routes of administration; changes in the vaccination schedule; the inclusion of potent adjuvants; the optimization of the antigen dose, etc. One of the reasons of their failure may be the fact that most of these clinical trials were conducted in patients under a deep and long-standing viral suppression [3–5].

The rationale of therapeutic vaccination when administered under viral suppression, is based on the observation that a decrease in viral load often precedes the detection of anti-HBV-specific T-cell responses, both in patients resolving natural infections and in those achieving HBeAg seroconversion during the chronic infection. Thus, a reduction in HBV load by antiviral treatment with nucleot(s)ide analogs (NUC) may increase the responsiveness of HBV-specific T cells, which are hyporesponsive in cases of persistent HBV infection [6]. However, changes in viral load have immunological repercussion.

Increased HBV-specific T-cell responses are detectable during the first few months of lamivudine treatment [7]; however, this restoration of T-cell activity is partial, transient and does not lead to an increase in HBeAg seroconversion [8], suggesting that the timing for vaccination is a relevant variable. On the other hand, the patients restarted NUC treatment very soon after discontinuation, just after the first detection of HBV DNA, or when there was evidence of HBV replication at low levels [9–11], following a conservative approach.

There are some theoretical disadvantages related to immunizing patients under long-term antiviral treatment. Vaccine-induced T cells should exert their function in the liver. However, the anti-inflammatory liver environment is reinforced, as reflected by the reduction in alanine amino transferase (ALT) levels in most patients few weeks after the start of antiviral treatment [9–11]. In line with this, it has been demonstrated that hepatocytes do not express HLA class II, except under inflammatory conditions [12–14], a logical and natural adaptation to the tolerogenic role of the liver, further reducing the antigenicity of hepatocytes in patients without treatment.

The suppression of viral replication in patients under NUC therapy leads to a reduction in the number of hepatocytes presenting viral antigens, such as cytoplasmic HBcAg. It has been demonstrated that the control of the replication can be predicted by the low intracellular expression of HBcAg [15]. Taken together, in virally suppressed patients, there is a reduced presentation of HBV antigens and consequently a reduction in the presentation of viral peptides to vaccine-induced T cells both by HLA class I and II. Viral replication reactivates the immune system detection mechanisms, restarting the immune response. In fact, nucleo(s/t)ide analog (NUC) treatment discontinuation may be useful for immunotherapeutic efficacy improvement; however the design of the discontinuation and restart treatment protocol needs to be changed.

HeberNasvac, a therapeutic vaccine used as a monotherapy, was compared with pegylated interferon (PegIFN) in an efficacy Phase III clinical trial [16]. This product was shown to be safer than PegIFN and more effective in terms of HBV DNA suppression. HeberNasvac also demonstrated a better serological response and a slower progression of fibrosis, linked to a lower number of complications in the 5-year follow-up period when compared with PegIFN [17–21]. It is noteworthy that the recent, long-lasting studies comparing PegIFN with antivirals support the superiority of PegIFN for the prevention of unfavorable events (cirrhosis, hepatocellular carcinoma, death) [22], even when the suppression of HBV is less pronounced compared with the effect of antiviral treatment. In this context, HeberNasvac's superiority compared with PegIFN deserves a closer look by WHO and other international organizations due to the safety profile of this product, having the potential of being applied to all chronic hepatitis B patients.

The sanitary registration granted to HeberNasvac in its country of origin (Cuba) as well as the first international activities with this product in Japan [23] and other countries in Asia, are encouraging news. HeberNasvac represents a finite and safe alternative for CHB treatment as a monotherapy. In addition, smart combinations with NUCs or PegIFN are also possible since it is a fully developed industrial product and, at present, administered in several hundreds of patients around the world as well as starting sanitary registration in several countries [24].

## Future opportunities in the field of therapeutic vaccination

There is still a long road ahead in the optimization of this product. In fact, the first clinical evaluation of HeberNasvac combined with NUC failed to demonstrate the expected efficacy according to the trial design [25,26]. Therapeutic vaccination combined with some of the novel therapeutic drugs under development [26] represents a wide opportunity for future research. However, the study of smart combinations with NUC treatment may overcome the immunological limitations found in past studies.

Major associations for the study of the liver support antiviral treatment discontinuation under strict monitoring in noncirrhotic HBeAg-negative patients, with long-time virological suppression and low fibrosis. The recommendations of treatment discontinuation in HBeAg-negative patients was preceded by a strong meta-analysis that detected the increase of the anti-HBV immune response after NUC cessation, as a consequence of viral rebound. ALT increases, in patients with low levels of fibrosis and under strict assessment, are considered benign in nature and reflect an immune activation that leads to HBsAg elimination in 20–40% of the patients in the 3-year follow-up period. On the other hand, patients continuing treatment with NUCs evidenced no reduction in their serum HBsAg levels [27–30].

The European Association for the Study of the Liver (EASL) has established rules for restarting therapy based on the absence of response, or on a clear and sustained re-emergence of the ALT and HBV DNA. Transient and moderate ALT increases after viral rebound are considered physiologically normal. This is later naturally controlled by the immune system. After a solid meta-analysis [27], EASL guidelines proposed the restart of NUC treatment, several months after discontinuation and specific rules were put in place.

The currently used clinical trial methodology should be adjusted to implement the novel guidelines proposed in patients discontinuing antiviral treatment. Such modifications favor the incorporation of patients currently under NUC treatment and wishing to stop antiviral treatment after several years. A potential increase in serological responses is likely to occur in such conditions [27]. Hence, a new horizon of opportunities is created for therapeutic vaccination approaches.

A rational design of vaccination in the setting of NUC interruption may also contribute to find a solution for patients needing antiviral treatment cessation due to economic or medical reasons. In this group, ALT exacerbation post-treatment cessation is frequent. In fact, irregular medication with NUCs is one of the major causes of decompensation and acute on chronic liver failure [31]. Thus, the modulation in the pattern of immune response with the use of vaccination around or after treatment cessation, should modulate the immunological pattern associated with viral rebound and ALT exacerbation associated to decompensation, in favour of shorter and beneficial flares related to the Th1 immune response induced by the vaccine [32].

An approach already under study proposes the use of NUC for a longer period of time after therapeutic vaccination. This approach is intended to favor HBsAg seroconversion without losing the virological, serological and biochemical gain from NUC therapy. In the specific case of HBeAg-negative patients under NUC, HBsAg remains as the only serological variable. The accomplishment of this concept represents a new strategy; in short, more time is given to the immune system to work under virological suppression [23]. The results of this new trial should provide light on this novel approach, leading to NUC treatment discontinuation only after the consolidation of HBsAg elimination.

In summary, therapeutic vaccination has the potential to modify immune reactivation after treatment cessation. Alternatively, the use of NUC for a longer period of time after therapeutic vaccination should favor HBsAg seroconversion without losing the virological, serological and biochemical gain from NUC therapy. Then, the future of therapeutic vaccination in CHB remains alive and strong, both as a monotherapy and in the setting of rationally combined treatments.
